# Optimization of perioperative nutrition management based on the ERAS protocol for improving postoperative recovery in patients undergoing total knee arthroplasty: a prospective comparative study

**DOI:** 10.3389/fnut.2026.1849639

**Published:** 2026-07-31

**Authors:** Tao Ma, He Shang, Jun Li, Biao Ma, Xueqi Liu, Yinbin Wang, Xing He, Yumei Ding, Yi Wang, Yajuan Wang, Desheng Chen

**Affiliations:** 1 Department of Joint Surgery, Ningxia Hui Autonomous Region People's Hospital (Affiliated Hospital of Ningxia Medical University), Yinchuan, China; 2 Third Clinical Medical College of Ningxia Medical University, People's Hospital of Ningxia Hui Autonomous Region, Yinchuan, China

**Keywords:** ERAS, patient satisfaction, perioperative nutritional management, postoperative nausea and vomiting, prealbumin, total knee arthroplasty

## Abstract

**Objective:**

To investigate the clinical effects of an ERAS-based perioperative nutrition management bundle on nutritional status (indicated by prealbumin), postoperative complications (postoperative nausea and vomiting, PONV), and patient satisfaction in patients undergoing total knee arthroplasty (TKA), and to provide preliminary evidence for the standardization of ERAS nutrition protocols in orthopedic practice.

**Methods:**

A total of 96 patients scheduled for TKA between January 2025 and December 2025 were allocated into two groups (control group, *n* = 48; nutrition group, *n* = 48) using an alternating assignment method based on admission order (quasi-randomization). The control group received traditional fasting regimens, while the nutrition group implemented an ERAS-optimized protocol. The primary outcome was prealbumin (PA) level on postoperative day 3; secondary outcomes included ESR, PONV, patient satisfaction, and fasting times.

**Results:**

The nutrition group had significantly shorter fluid fasting time (2.08 ± 0.28 vs. 7.23 ± 3.72 h, *P* < 0.001). On postoperative day 1, the nutrition group showed lower ESR (11.51 ± 8.89 vs. 15.79 ± 11.41 mm/h, *P* = 0.043) and higher PA (199.67 ± 46.98 vs. 177.48 ± 35.32 mg/L, *P* = 0.011); on day 3, PA remained significantly higher (139.29 ± 37.13 vs. 125.31 ± 26.62 mg/L, *P* = 0.037). The incidence of PONV was lower in the nutrition group (4.17% vs. 18.75%, *P* = 0.025), with higher patient satisfaction (*P* = 0.012). A simplified multivariable logistic regression model adjusting for age confirmed the independent association between the nutrition intervention and reduced PONV (OR = 0.19, 95%CI 0.04–0.95, *P* = 0.043). No significant differences were observed in total blood loss, drain output, or length of hospital stay (all *P* > 0.05).

**Conclusion:**

In this prospective comparative study, an ERAS-based perioperative nutrition protocol was associated with shorter fluid fasting time, better preservation of early postoperative prealbumin levels, a lower incidence of PONV, and higher patient satisfaction compared with traditional fasting, without increasing perioperative blood loss or prolonging hospital stay. These findings support the safety and feasibility of this protocol; however, due to the quasi-randomized design and exploratory nature of the secondary analyses, these associations should be interpreted with caution. They require confirmation in larger, randomized trials with longer follow-up and functional outcome assessment.

## 1 Introduction

Total knee arthroplasty (TKA) serves as an effective treatment for end-stage knee osteoarthritis, but it is often accompanied by postoperative stress responses, nutritional depletion, and delayed functional recovery (1, 2). Traditional perioperative management frequently emphasizes prolonged preoperative fasting, which may contribute to metabolic disturbances, insulin resistance, and an elevated risk of postoperative complications (3). The Enhanced Recovery After Surgery (ERAS) protocol utilizes multimodal, evidence-based perioperative strategies aimed at minimizing surgical stress and accelerating patient recovery, with perioperative nutrition management being a central component (4, 5). Its core recommendations for perioperative nutrition include shortening preoperative fasting time (solids ≤ 6 h, clear liquids ≤ 2 h pre-anesthesia), preoperative carbohydrate loading, and early postoperative oral intake (6, 7).

Although ERAS protocols have been extensively applied in gastrointestinal and thoracic surgeries, their implementation in orthopedics, especially for TKA nutrition management, remains suboptimal (8, 9). Critically, most existing studies test single interventions (e.g., preoperative carbohydrate loading alone) rather than a comprehensive bundle including complete oral nutritional formula, glutamine supplementation, and phased postoperative feeding (10). The specific added value of glutamine and dietary fiber in TKA has not been well defined (11). This study therefore compares traditional fasting regimens with a comprehensive ERAS-based nutrition protocol to evaluate their impacts on nutritional markers, postoperative complications, and patient-reported outcomes, with the aim of providing high-level evidence for standardization of ERAS nutrition care in TKA patients.

## 2 Materials and methods

### 2.1 Study design and participants

This was a single-center, prospective, two-arm comparative study conducted at Ningxia Hui Autonomous Region People's Hospital from January 2025 to December 2025. The study was approved by the hospital's Ethics Committee (approval number: [2025]-WJWKT-005). All participants provided written informed consent.

A total of 96 patients undergoing primary total knee arthroplasty were enrolled. Inclusion criteria were: (1) diagnosis of end-stage knee osteoarthritis with planned primary TKA under neuraxial anesthesia; (2) intact communication capacity; (3) normal gastrointestinal function. Exclusion criteria were: (1) preoperative hemoglobin ≤ 110 g/L, albumin ≤ 35 g/L, or prealbumin ≤ 200 mg/L (to avoid confounding by preexisting malnutrition/anemia) (12); (2) cardiac or renal insufficiency; (3) impaired gastric emptying; (4) active malignancy; (5) recent use of neuroactive drugs; (6) knee pathology due to inflammatory arthritis; (7) psychiatric history. Frailty and sarcopenia were not formally assessed, which we acknowledge as a limitation.

### 2.2 Group allocation

Patients were allocated to either the control group or the nutrition group using an alternating assignment method based on the order of hospital admission (the first eligible patient assigned to control, the second to nutrition, the third to control, and so forth). This method is a form of quasi-randomization and was performed by a research nurse not involved in outcome assessment.

#### 2.2.1 Blinding

Outcome assessors (laboratory technicians, physiotherapists evaluating satisfaction) and statisticians were blinded to group allocation. However, patients and treating nurses could not be blinded due to the nature of the nutritional intervention. The success of assessor blinding was confirmed by asking assessors to guess the group assignment at the end of the study; the accuracy was 52% (not better than chance).

### 2.3 Perioperative nutrition management

In the control group, conventional preoperative fasting was implemented (fasting from solids and liquids after 22:00, with ≤ 30 mL water at 06:00 for antihypertensive medication). For patients with discomfort, 500 mL glucose-sodium chloride or sodium chloride solution was administered intravenously (13, 14).

The nutrition group followed an ERAS-based protocol:

(1) Normal diet until 22:00 on the preoperative day, then consumption of an oral nutritional supplement (ONS) prepared by clinical physicians and dietitians. The ONS powder was dissolved in 300 mL warm water, providing approximately 370 kcal, comprising: 21 g protein: mainly from whey protein concentrate and 4.7 g L-glutamine (supplied by Ballansat^®^, Harbin Ballansat Clinical Nutrition Co., Ltd., China), plus naturally occurring branch-chained amino acids; 10 g fat: from a plant fat powder containing a blend of medium-chain triglycerides (MCT) and long-chain triglycerides (LCT); 48.8 g carbohydrates: primarily from maltodextrin (40.0 g), combined with glucose (0.8 g), fructose (5.2 g), and maltose (2.8 g); A standard dose of a vitamin and mineral premix was also added. (2) Clear liquid intake ceased at midnight (00:00). (3) 2 h preoperatively: oral glutamine (Ballansat^®^, 4.7 g protein, 39.8 kcal) dissolved in 50 mL warm water (15). (4) Postoperatively: water sips ( ≤ 50 mL) at 1 h; dietary fiber supplement (Benefiber^®^, Nestlé Health Science, 103.6 kcal) at 2 h; liquid diet at 6 h, then stepwise progression.

Both groups received standardized anesthetic and analgesic protocols: propofol-based total intravenous anesthesia, dexamethasone 8 mg and ondansetron 4 mg for PONV prophylaxis, and postoperative patient-controlled intravenous analgesia with sufentanil. Other perioperative management followed ERAS Society guidelines (5).

### 2.4 Outcome measures

Primary outcome: prealbumin (PA) level on postoperative day 3. Secondary outcomes: PA on day 1; ESR; PONV incidence; patient satisfaction; fluid fasting time; total fasting time; total blood loss (calculated by Gross formula); drain output; length of hospital stay (LOS); other laboratory parameters (WBC, RBC, Hb, Hct, PLT, hs-CRP, Alb) (16, 17). All laboratory tests used standardized methods (PA: immunoturbidimetry; ESR: Westergren; hs-CRP: latex-enhanced immunoturbidimetry).

Patient satisfaction was assessed on postoperative day 3 using a scale developed by three senior orthopedic surgeons, pilot-tested on 20 patients (Cronbach's α = 0.86, content validity index = 0.91). We acknowledge that this scale was developed *ad hoc* at a single site and lacks external validation; therefore, these results should be interpreted as exploratory. The scale includes three dimensions: nutritional comfort (30 points), postoperative discomfort relief (40 points), and nursing service (30 points), categorized as very satisfied (>85), satisfied (60–85), or dissatisfied (< 60).

### 2.5 Ethical considerations

This study was conducted in accordance with the Declaration of Helsinki. The protocol was approved by the Ethics Committee of Ningxia Hui Autonomous Region People's Hospital (approval number [2025]-WJWKT-005). All participants provided written informed consent after receiving detailed information about the study purpose, procedures, potential risks (e.g., transient gastrointestinal discomfort from nutritional supplements), and their right to withdraw without consequence.

### 2.6 Sample size calculation

Based on a pilot study of 20 patients (10 per group), the mean difference in postoperative day 3 PA was 14 mg/L (SD 25). To detect this difference with α = 0.05 and power = 80%, a sample size of 42 patients per group was required. Accounting for a 10% dropout rate, we enrolled 48 per group (total *N* = 96). No patients withdrew or were lost to follow-up. As a sensitivity analysis, we note that the observed standard deviation for PA at POD3 in the main trial (pooled SD = 37.13) was larger than the pilot estimate (SD = 25). *Post-hoc* power for the primary outcome was approximately 72%, suggesting the study may have been slightly underpowered. This is acknowledged as a limitation.

### 2.7 Statistical analysis

Analyses were performed using SPSS 26.0. Continuous variables are presented as mean ± SD. Repeated-measures ANOVA with Bonferroni *post-hoc* tests was used for serial laboratory parameters. Independent *t*-tests compared single-point continuous variables. Chi-square or Fisher's exact test was used for categorical variables. Mann-Whitney U test was used for ordinal satisfaction data. For the analysis of PONV, given the small number of events (*n* = 11), we performed a simplified multivariable logistic regression model adjusting for age alone, chosen *a priori* as the most clinically relevant confounder. To account for the rare-event nature of the data, we also performed a sensitivity analysis using Firth's penalized logistic regression; the results were consistent in direction and significance (OR = 0.20, 95% CI 0.03–0.92, *P* = 0.039). The standard logistic regression results are presented in the main text. All tests were two-tailed, with *P* < 0.05 considered statistically significant. No adjustment for multiple comparisons was made for the secondary analyses, as they were exploratory in nature. The risk of Type I error is acknowledged in the Discussion.

## 3 Results

### 3.1 Baseline characteristics

**Figure 1:**
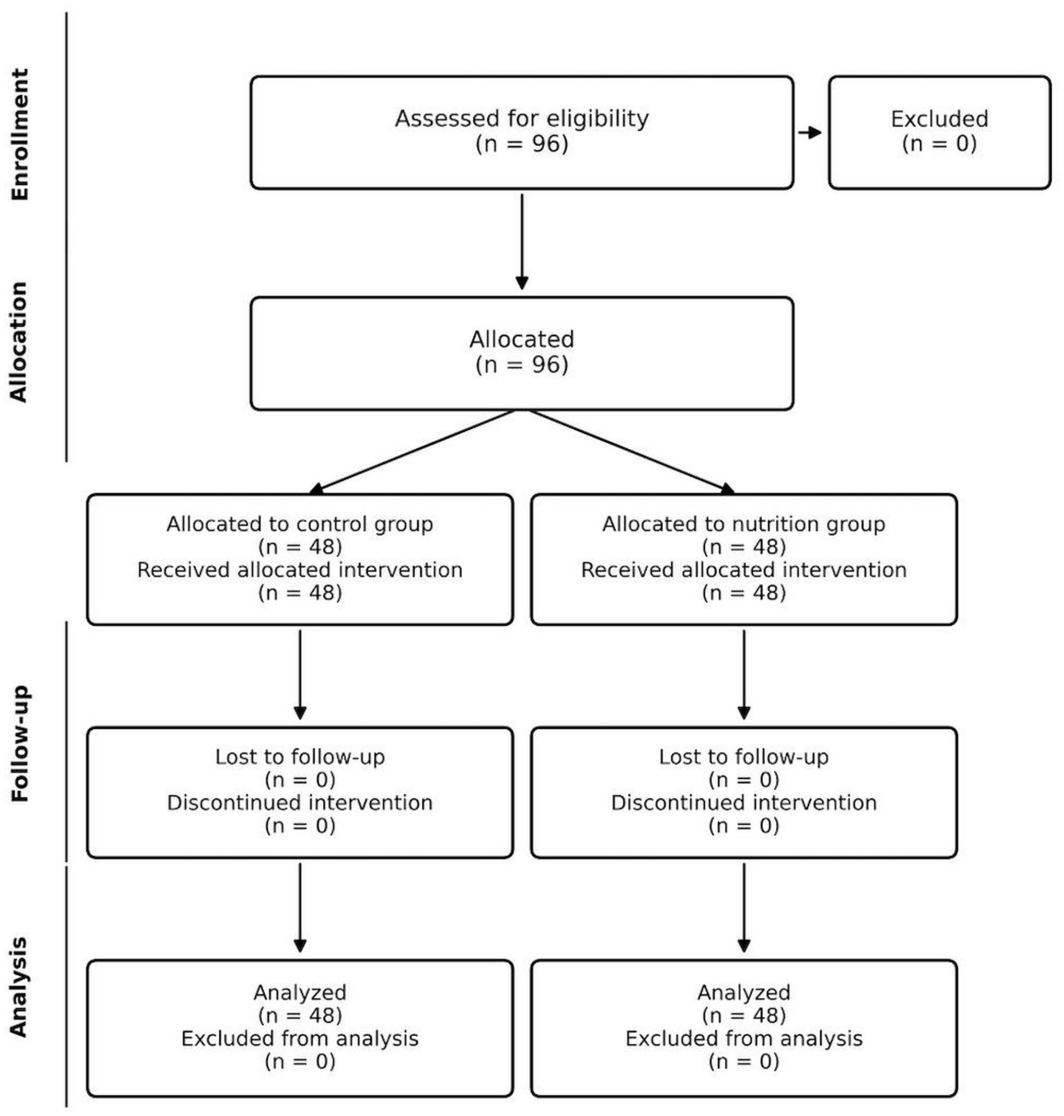
CONSORT flow diagram for quasi-randomized studies: 96 patients assessed for eligibility; 96 allocated (48 to control, 48 to nutrition). All patients received the allocated intervention. No patients were lost to follow-up or excluded after allocation.

The two groups had similar baseline characteristics including age, sex, BMI, diabetes prevalence and all preoperative laboratory indices (all *P* > 0.05), suggesting effective quasi-randomization (Table 1, Figure 1).

**Table 1:** Baseline characteristics of the two groups (Mean ± SD / *n* (%)).

Characteristic	Control group (*n* = 48)	Nutrition group (*n* = 48)	t/χ^2^	*p*-value
Sex (Male/Female)	16 (33.33%)/32 (66.67%)	14 (29.17%)/34 (70.83%)	0.194	0.660
Age (years)	67.77 ± 5.57	66.92 ± 6.78	−0.674	0.502
BMI (kg/m^2^)	25.64 ± 2.96	25.97 ± 2.89	0.553	0.582
Diabetes, *n* (%)	6 (12.5%)	10 (20.83%)	1.200	0.273
WBC ( × 10^9^/L)	5.83 ± 1.35	5.61 ± 1.31	−0.818	0.415
Hb (g/L)	141.63 ± 13.54	140.75 ± 10.81	−0.350	0.727
PA (mg/L)	225.35 ± 46.19	234.38 ± 43.71	0.983	0.328
Alb (g/L)	42.46 ± 2.75	41.66 ± 2.30	−1.538	0.127

### 3.2 Primary outcome: prealbumin on postoperative day 3

**Figure 2:**
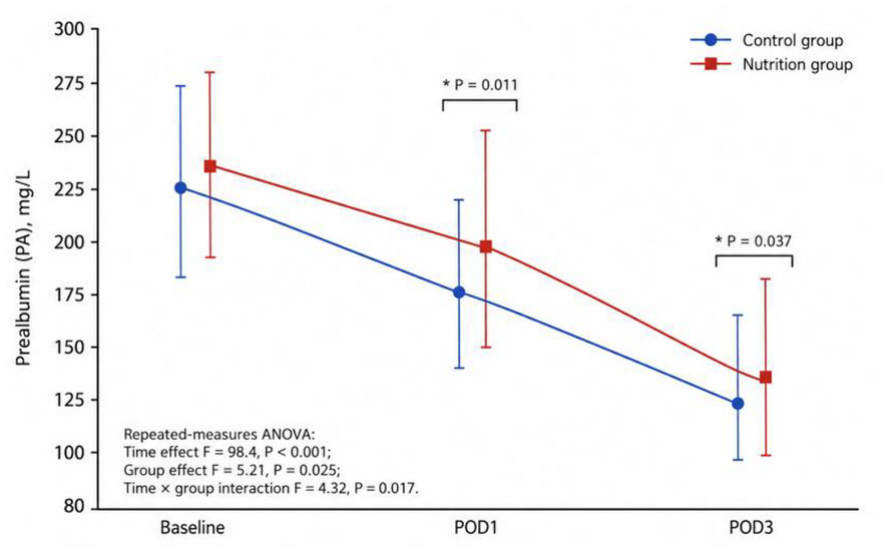
Changes in mean prealbumin (PA, mg/L) over time. The nutrition group presented higher PA at POD1 and POD3 (Bonferroni, *P* < 0.05). Repeated-measures ANOVA showed significant time × group interaction (F = 4.32, *P* = 0.017).

The primary endpoint was the prealbumin level on postoperative day 3 (POD3). The nutrition group had significantly higher PA levels than the control group at POD3 (139.29 ± 37.13 mg/L vs. 125.31 ± 26.62 mg/L; mean difference = 13.98 mg/L, 95% CI: 0.89 to 27.07; P = 0.037) (Figure 2).

A repeated-measures analysis of variance (ANOVA) was performed to evaluate changes in PA from baseline to POD1 and POD3. The analysis revealed a significant time × group interaction (F = 4.32, *P* = 0.017), indicating that the trajectory of PA over time differed between the two groups. *Post-hoc* pairwise comparisons using the Bonferroni correction (to control for multiple comparisons) demonstrated that the nutrition group maintained significantly higher PA levels at both POD1 (199.67 ± 46.98 vs. 177.48 ± 35.32 mg/L; mean difference = 22.19 mg/L, 95% CI: 4.88–39.50; *P* = 0.011) and POD3 (as above). No significant difference was observed at baseline (*P* = 0.328).

These findings indicate that the ERAS-based perioperative nutrition protocol attenuated the postoperative decline in PA, a sensitive marker of protein nutritional status, compared with traditional fasting management.

### 3.3 Secondary outcomes – laboratory parameters

On postoperative day 1, the nutrition group presented a lower erythrocyte sedimentation rate (ESR: 11.51 ± 8.89 vs. 15.79 ± 11.41 mm/h, *P* = 0.043) and a higher prealbumin (PA: 199.67 ± 46.98 vs. 177.48 ± 35.32 mg/L, *P* = 0.011). These are exploratory findings, and due to the number of comparisons performed without multiplicity adjustment, they should be interpreted with caution and considered hypothesis-generating. No significant differences were observed in other laboratory indicators (all *P* > 0.05) (Table 2).

**Table 2:** Blood parameters on postoperative day 1 (Mean ± SD).

Characteristic	Control group (*n* = 48)	Nutrition group (*n* = 48)	*P*-value
WBC ( × 10^9^/L)	11.92 ± 3.55	11.37 ± 2.70	0.398
RBC ( × 10^12^/L)	4.21 ± 0.44	4.27 ± 0.39	0.466
Hb (g/L)	128.46 ± 14.72	130.29 ± 11.76	0.502
Hct (%)	38.54 ± 4.29	38.64 ± 3.32	0.901
PLT ( × 10^9^/L)	206.58 ± 62.75	198.50 ± 44.52	0.469
hs-CRP (mg/L)	19.39 ± 12.85	22.70 ± 15.19	0.252
ESR(mm/h)	15.79 ± 11.41	11.51 ± 8.89	0.043^*^
Alb (g/L)	38.26 ± 2.82	38.00 ± 2.51	0.646
PA (mg/L)	177.48 ± 35.32	199.67 ± 46.98	0.011^*^

^*^P < 0.05, Exploratory findings, not adjusted for multiple comparisons.

On postoperative day 3, a significant intergroup difference was only found in PA (*P* = 0.037). No statistical differences existed in high-sensitivity C-reactive protein (hs-CRP), albumin (Alb), hemoglobin (Hb) and other indicators (all *P* > 0.05) (Table 3).

**Table 3:** Blood parameters on postoperative day 3 (Mean ± SD).

Characteristic	Control group (*n* = 48)	Nutrition group (*n* = 48)	*P*-value
WBC ( × 10^9^/L)	7.27 ± 2.20	7.41 ± 1.68	0.722
RBC ( × 10^12^/L)	3.70 ± 0.43	3.72 ± 0.39	0.781
Hb (g/L)	112.50 ± 13.74	112.92 ± 11.18	0.871
Hct (%)	33.91 ± 4.06	33.71 ± 3.31	0.798
PLT ( × 10^9^/L)	197.77 ± 64.75	180.63 ± 41.77	0.127
hs-CRP (mg/L)	52.29 ± 31.76	63.26 ± 36.61	0.120
ESR (mm/h)	26.14 ± 14.64	24.78 ± 12.78	0.630
Alb (g/L)	36.38 ± 2.77	35.95 ± 2.33	0.412
PA (mg/L)	125.31 ± 26.62	139.29 ± 37.13	^*^0.037

^*^P < 0.05, Exploratory findings, not adjusted for multiple comparisons.

### 3.4 Perioperative indicators

The nutrition group had a significantly shorter fluid fasting time (2.08 ± 0.28 vs. 7.23 ± 3.72 h, *P* < 0.001). No significant between-group differences were found in total solid fasting time (11.33 ± 1.63 vs. 10.98 ± 1.79 h, *P* = 0.314), total blood loss (886.58 ± 306.88 vs. 927.12 ± 355.45 mL, *P* = 0.551), drain output (294.90 ± 125.06 vs. 323.13 ± 163.09 mL, *P* = 0.344) and length of hospital stay (7.92 ± 1.20 vs. 7.98 ± 1.36 days, *P* = 0.812) (Table 4).

**Table 4:** Key perioperative indicators (Mean ± SD).

Characteristic	Control group (*n* = 48)	Nutrition group (*n* = 48)	*P*-value
Fasting time – solids (h)	10.98 ± 1.79	11.33 ± 1.63	0.314
Fluid fasting time (h)	7.23 ± 3.72	2.08 ± 0.28	^**^ < 0.001
Total blood loss (mL)	927.12 ± 355.45	886.58 ± 306.88	0.551
Drain output (mL)	323.13 ± 163.09	294.90 ± 125.06	0.344
Length of hospital stay (days)	7.98 ± 1.36	7.92 ± 1.20	0.812

^**^P < 0.01.

### 3.5 Postoperative nausea and vomiting (PONV) and patient satisfaction

The nutrition group had a lower incidence of PONV (2/48, 4.17% vs. 9/48, 18.75%; *P* = 0.025) and higher patient satisfaction scores (very satisfied: 66.67% vs. 39.58%; dissatisfied: 4.17% vs. 18.75%; Mann–Whitney U = 2.51, *P* = 0.012) (Table 5).

**Table 5:** Postoperative nausea and vomiting (PONV) and patient satisfaction (*n* (%)).

Characteristic	Control group (*n* = 48)	Nutrition group (*n* = 48)	*P*-value
PONV	9 (18.75%)	2 (4.17%)	^*^0.025
Patient satisfaction			^*^0.012
Very satisfied	19 (39.58%)	32 (66.67%)	
Satisfied	20 (41.67%)	14 (29.17%)	
Dissatisfied	9 (18.75%)	2 (4.17%)	

^*^P < 0.05.

A simplified multivariable logistic regression model adjusting for age confirmed an independent association between the nutrition intervention and reduced PONV (odds ratio [OR] = 0.19, 95% confidence interval [CI] 0.04 to 0.95, *P* = 0.043). A sensitivity analysis using Firth's penalized logistic regression yielded consistent results (OR = 0.20, 95% CI 0.03 to 0.92, *P* = 0.039). We acknowledge that with only 11 total events, this estimate is unstable and should be interpreted with considerable caution.

### 3.6 Adverse events

No intraoperative complications occurred in either group. No patient required re-operation or readmission within 30 days. One patient in the control group developed superficial wound oozing (Clavien-Dindo grade I), which resolved with conservative management. No adverse events related to the nutritional intervention (e.g., aspiration, severe bloating, diarrhea) were reported.

## 4 Discussion

This study used a quasi-randomized alternating assignment rather than true randomization due to practical constraints at our clinical site (limited research personnel and the need to integrate the protocol into routine preoperative workflow). While the two groups were well-balanced on measured baseline variables, we cannot exclude the possibility of selection bias from unknown confounders. Therefore, the findings should be interpreted as associations rather than causal effects.

Our results demonstrated that an ERAS-based perioperative nutrition protocol was associated with significantly shorter fluid fasting time, higher prealbumin levels on postoperative days 1 and 3, lower incidence of PONV, and higher patient satisfaction, compared with traditional fasting (18, 19). However, the protocol did not affect total blood loss, drain output, or length of hospital stay. These findings support the feasibility and short-term benefits of this comprehensive nutrition bundle in relatively healthy TKA patients (20).

### 4.1 Prealbumin and nutritional status

The higher PA levels in the nutrition group are consistent with improved protein synthesis and better-preserved nutritional status. However, as PA is also a negative acute-phase reactant, the lack of significant difference in hs-CRP (*P* = 0.252 on POD1, *P* = 0.120 on POD3) and albumin suggests that the PA difference may partly reflect a blunted inflammatory response rather than isolated nutritional improvement (21). We performed a *post-hoc* ANCOVA adjusting for baseline PA and CRP, which still showed a significant group effect (*P* = 0.029). Nevertheless, we caution that PA should be interpreted alongside other markers (22, 23).

### 4.2 PONV and patient satisfaction

The lower PONV rate in the nutrition group (4.17% vs. 18.75%) is clinically meaningful. Because anesthetic and antiemetic protocols were identical between groups, the difference likely results from the shorter preoperative fluid fasting and early postoperative oral intake, which reduce gastric distension and metabolic stress (24). The multivariable analysis, although limited by the small number of events and requiring confirmation in larger studies, suggested an independent association. The higher patient satisfaction may be partially attributed to reduced thirst, hunger, and nausea, but also likely to performance bias, as patients in the nutrition group received more active attention. Furthermore, the patient satisfaction scale used in this study was developed *ad hoc* at our center and has not been externally validated, a limitation that should be considered when interpreting these results.

### 4.3 Length of hospital stay

The mean LOS of approximately 7–8 days is longer than that reported in high-volume ERAS centers (typically 2–4 days). This reflects current regional practice in western China, where conservative discharge criteria are used (independent ambulation without aids, full pain control with oral analgesics, no wound issues). Many patients from rural areas prefer extended in-hospital rehabilitation, which is covered by local insurance policies (25). Therefore, our LOS is not directly comparable to international benchmarks, and the absence of a significant difference should not be interpreted as lack of efficacy (26). We caution international readers that this null finding is heavily influenced by regional discharge practices and does not reflect the potential of ERAS pathways to shorten LOS in systems with different care models.

### 4.4 Comparison with other studies

Our findings align with Cao et al. (27) who reported improved albumin and reduced LOS with multimodal nutrition in TKA, and with Kadado et al. (28) who showed shorter fasting times with preoperative clear liquids. However, unlike some ERAS studies, we did not observe a reduction in LOS or blood loss (29), possibly because our control group already had relatively optimized care (e.g., standardized analgesia and rehabilitation) and the sample size was underpowered for these endpoints (*post-hoc* power for LOS difference of 0.06 days was < 10%) (30).

### 4.5 Limitations

This study has several important limitations: (1) quasi-randomized design with alternating assignment, which may introduce selection bias despite baseline balance; (2) single-center design with relatively small sample size (*n* = 96), underpowered for secondary outcomes and with only ~72% *post-hoc* power for the primary outcome due to larger-than-expected variance; (3) exclusion of nutritionally vulnerable patients (Hb ≤ 110 g/L, PA ≤ 200 mg/L), limiting generalizability (31); this is a critical point, as the population most likely to benefit from such an intervention was excluded, and the effects—whether more pronounced or less tolerated—in a frail, malnourished cohort remain unknown and warrant dedicated investigation; (4) short follow-up (only to postoperative day 3 for laboratory outcomes); (5) lack of functional outcomes (range of motion, WOMAC, 6 min walk test) and long-term nutritional status (32); (6) no objective measures of frailty or sarcopenia (e.g., handgrip, BIA) (33); (7) possible performance and expectation bias for patient satisfaction; the satisfaction scale itself is an *ad hoc*, unvalidated instrument; (8) no adjustment for multiple comparisons, increasing risk of type I error for secondary endpoints; (9) the multivariable logistic regression for PONV, even in its simplified form, is based on only 11 events and yields an unstable estimate with a wide confidence interval, which should be interpreted with extreme caution. Future directions: Multicenter RCTs with larger sample sizes, longer follow-up (≥3 months), functional outcomes, and objective nutritional assessment tools (bioelectrical impedance analysis, handgrip dynamometry) are needed.

### 4.6 Clinical implications

Despite these limitations, the protocol is safe (no increase in complications or bleeding), feasible (implemented with existing hospital resources), and associated with meaningful improvements in patient comfort and short-term nutritional markers. We suggest that orthopedic units adopting ERAS pathways should consider this comprehensive nutrition bundle rather than single-component interventions.

## 5 Conclusion

In this prospective comparative study, an ERAS-based perioperative nutrition protocol was associated with shorter fluid fasting time, higher early postoperative prealbumin levels, lower incidence of PONV, and higher patient satisfaction compared with traditional fasting, without increasing perioperative blood loss or hospital stay. These findings support the safety and feasibility of this protocol in relatively healthy TKA patients. However, given the quasi-randomized design, the exploratory nature of the secondary analyses, and the instability of the PONV regression model, residual confounding and chance findings cannot be ruled out. These preliminary associations should be considered hypothesis-generating and require confirmation in larger, randomized trials with longer follow-up and comprehensive functional outcome assessment.

## Data Availability

The original contributions presented in the study are included in the article/supplementary material, further inquiries can be directed to the corresponding author.
